# Fixation Strategy Influences the Ability to Focus Attention on Two Spatially Separate Objects

**DOI:** 10.1371/journal.pone.0065673

**Published:** 2013-06-11

**Authors:** Stefanie Hüttermann, Daniel Memmert, Daniel J. Simons, Otmar Bock

**Affiliations:** 1 Institute of Cognitive and Team/Racket Sport Research, German Sport University Cologne, Cologne, Germany; 2 Department of Psychology, University of Illinois, Champaign, Illinois, United States of America; 3 Institute of Physiology and Anatomy, German Sport University Cologne, Cologne, Germany; CNRS - Université Claude Bernard Lyon 1, France

## Abstract

The ability to devote attention simultaneously to multiple visual objects plays an important role in domains ranging from everyday activities to the workplace. Yet, no studies have systematically explored the fixation strategies that optimize attention to two spatially distinct objects. Assuming the two objects require attention nearly simultaneously, subjects either could fixate one object or they could fixate between the objects. Studies measuring the breadth of attention have focused almost exclusively on the former strategy, by having subjects simultaneously perform one attention-demanding task at fixation and another in the periphery. We compared performance when one object was at fixation and the other was in the periphery to a condition in which both objects were in the periphery and subjects fixated between them. Performance was better with two peripheral stimuli than with one central and one peripheral stimulus, meaning that a strategy of fixating between stimuli permitted greater attention breadth. Consistent with the idea that both measures tap attention breadth, sport experts consistently outperformed novices with both fixation strategies. Our findings suggest a way to improve performance when observers must pay attention to multiple objects across spatial regions. We discuss possible explanations for this performance advantage.

## Introduction

Many real-life situations require people to focus attention on two locations simultaneously. Drivers devote attention both to their own car and to the traffic around them, pedestrians focus on their path while avoiding obstacles [1], cooks devote attention to multiple pots and ingredients, athletes focus on the ball and on the opposing player [2], and lifeguards monitor children playing in different locations. Despite the ubiquitous need to distribute attention across multiple objects in our daily behavior, relatively few studies (e.g., [3], [4], [5], [6]) have examined the strategies people use when devoting attention to multiple objects simultaneously, and even fewer (e.g., [7]) have explored individual differences in the breadth of visual attention across these situations.

In many cases, observers can shift attention from one object to another, often by fixating each sequentially. However, when people must attend to both nearly simultaneously [8], they have two primary options: They can fixate one object and attend to the other in the periphery or they can fixate between the objects and attend to both in the periphery [9]. Only the first option has been studied systematically.

In the Useful Field of View (UFOV) task, observers perform two concurrent monitoring tasks, one at fixation and the other in the periphery [10], [11], [12], [13]. The task provides a measure of attention breadth based on the visual eccentricity at which subjects can detect the peripheral target. The UFOV is typically smaller than the visual field (as determined by perimetry), decreases in old age, and predicts impaired driving, traffic accidents, and traffic citations in elderly drivers (e.g., [14], [15], [16], [17], [18], [19], [20]).

Although the UFOV provides a reliable and valid measure of attention breadth, it tests only the situation in which observers perform one task at fixation and detect targets in the periphery: It does not address cases in which both targets are peripheral and it does not equate the two task components for their demands on attention. In the UFOV, the fixation task demands sustained focused attention, but the peripheral task just requires detection. In many real-life situations (e.g., in driving or sports), though, both locations demand attention equally. And, fixating one target and attending to the other peripherally might not be the optimal performance strategy.

To examine performance with different strategies, we created a new “attention breadth” task in which observers must attend to two equally attention demanding stimuli simultaneously to respond correctly. In the “Fixate Center” condition participants focus their gaze between two stimuli. In the “Fixate Target” condition, participants focus their gaze on one stimulus and process the other peripherally.

The Fixate Target condition is conceptually similar to the UFOV task in which people fixate one set of stimuli and detect another peripherally. Unlike the UFOV, though, the stimuli and task for the central and peripheral stimuli are identical, and both demand attention because they require a conjunction search [21], [22]. Moreover, the stimuli and tasks are identical across our two conditions, making the results directly comparable.

We predicted that people would be better able to process two stimuli simultaneously when fixating between them than when fixating on one of them. More precisely, we predicted that they would successfully perform the task with greater separation between the stimuli when fixating between the targets, for two reasons: First, with the same separation between the targets, each target is closer to fixation in the Fixate Center condition than is the single peripheral target in the Fixate Target condition. Given that both stimuli require the same extent of focused attention, fixating between them will allow participants to split attention to them equally. Second, the ability to attend both to stimuli at fixation and to stimuli in the periphery might be more effortful because fixation receives priority by default (e.g., see [23]). Given that both stimuli place the same demands on attention, prioritizing one of them could lead to inferior overall performance when subjects must respond to both in order to achieve correct performance.

We validated our measures of attention by comparing expert and novice team athletes who play sports that require them to focus on multiple objects simultaneously. Athletes distribute attention more effectively over multiple locations than do non-athletes, they switch their attention more rapidly among locations [24], and they can maintain attention longer than novices [25], [2], [26], [27], [28]. These advantages should lead to greater attention breadth for expert athletes in both of our tasks as well.

## Methods

### Participants

Participants were ten expert athletes (6 female, *M*_age_ = 24.60, *SD* = 2.46 years) with more than ten years of intensive training as players in a team sport (*M*_team sports experience_ = 13.40, *SD* = 2.37 years; [29]) and nine relative novices (5 female, *M*_age_ = 26.33, *SD* = 3.50 years, *M*_team sports experience_ = 4.11, *SD* = 3.14 years). The current team sport disciplines of our subjects were handball (*n* = 2), hockey (*n* = 2), soccer (*n* = 11), volleyball (*n* = 2), or no current sport (*n* = 2, both novices). All subjects reported normal vision without need for corrective lenses, and none had participated in sensorimotor research within the preceding six months. The study was approved by the Ethics board of the German Sport University Cologne. Written informed consent was obtained from each subject prior to participation in the study in accordance with the principles of the Declaration of Helsinki 1975.

### Materials and Procedure

**Figure 1:**
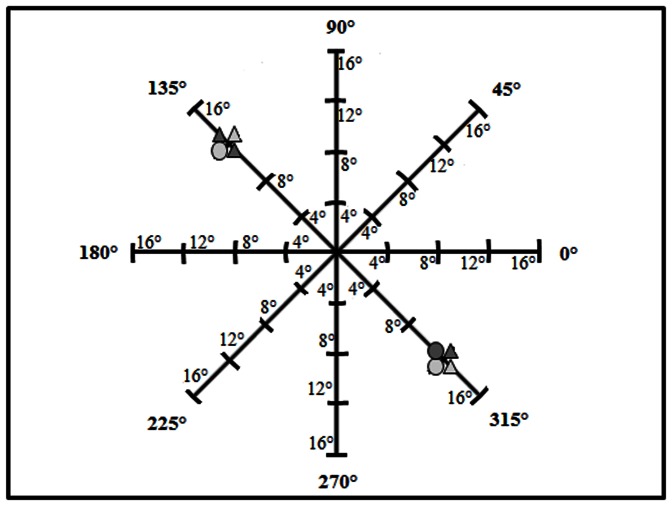
The stimuli were located at one of four distances from the center of the screen along one of four meridians (0°/180°, 45°/225°, 90°/270°, or 135°/315°). The figure shows a stimulus pair separated by 24° along the 135°/315° meridian. Note that the meridians and distance markings were not visible to participants and are included here only for illustration.

Subjects participated first in a Control condition, followed by the Fixate Target and Fixate Center conditions, with the order of the two attention breadth conditions counterbalanced across subjects. They sat approximately 2.70 m from a 2.80 m×2.20 m white projection screen with a visual angle of 55° in the horizontal and 44° in the vertical direction. Stimuli generated with E-Prime® were presented at one of four distances from the center of the screen (4°, 8°, 12°, or 16° of visual angle away from the center) along one of four meridians (see Figure 1).

The Control condition was designed to verify that performance in the primary condition was limited by attention and not by visual acuity. Participants were asked to report the properties of a single stimulus that appeared at various distances from fixation. In the attention breadth conditions, two such stimuli appeared equidistant from and on opposite sides of the center of the screen along one of the meridians. (Figure 1 shows a stimulus pair located on the 135°/315° meridian with each stimulus from the screen center was 12°, resulting in a total visual angle between the stimuli of 24°.) Stimulus positions were selected at random from the set of 32 possible locations in the Control condition and from the set of 16 pairs of possible locations on each of the 192 trials of each attention breadth condition. Trials were equally divided among the three meridian types (one third along the horizontal, one third along the vertical, and the remaining third equally dived across the two diagonal meridians). Each stimulus separation appeared equally often along each meridian type.

Each stimulus subtended 19×19 cm (equal to 4.03°), and consisted of four 9×9 cm (equal to 1.91°) elements arranged in a square with a gap of 1 cm (equal to 0.21°) between the elements. Each element had one of two shapes (circle, triangle) and one of two shades (light gray, dark gray), giving four possible element types. The subjects’ task was to verbally report the number of light gray triangles in each stimulus. Each stimulus could include 0, 1, 2, 3, or 4 light gray triangles with equal probability (20% of trials for each number in each condition), with stimulus types randomly assigned on each trial. Each attention breadth condition was preceded by 16 practice trials, and subjects were given a 30 s break after every 64 trials.

The Control condition verified that the targets remained visible at all eccentricities when observers only needed to focus attention on one stimulus. Any decrement in performance with two stimuli could then be attributed to the limits of attention rather than the limits of acuity. Each trial in the Control condition began with a central fixation cross (1000 ms), followed by a 200 ms pre-cue circle (8 cm diameter, equal to 1.70°) indicating the future location of the target (in one of the 32 possible locations). After a 200 ms blank interval, the target appeared for 150 ms. A prompt then asked subjects for an un-speeded verbal report of the number of light gray triangles in the stimulus. Subjects were reminded to keep their gaze on the fixation cross throughout each trial and to identify the stimulus using peripheral vision.

**Figure 2:**
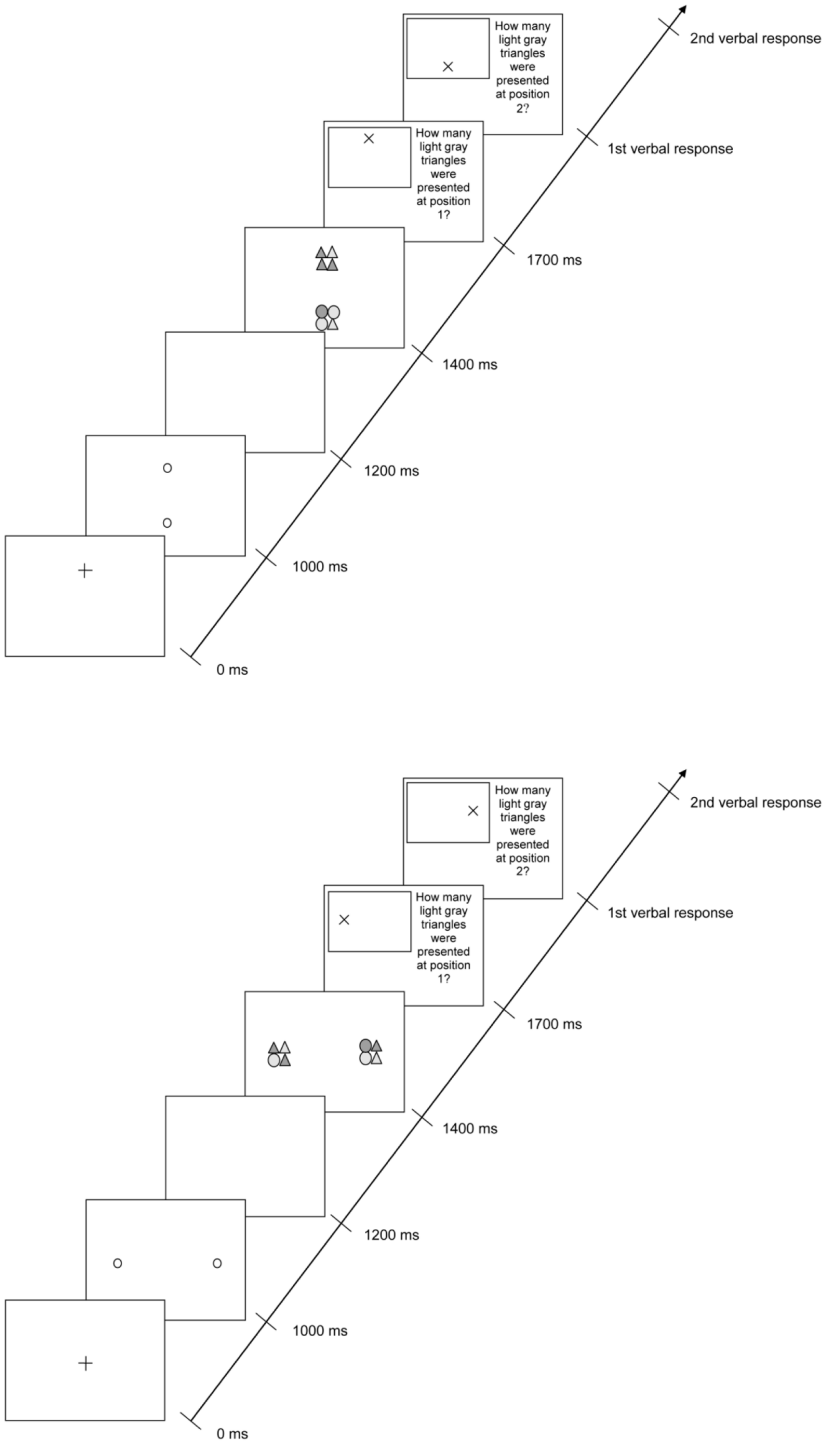
Sequence of events in one trial from the Fixate Target condition (top panel) and the Fixate Center condition (bottom panel). In the top panel, the two stimuli appear on the 90°/270° meridian. In the bottom panel, the two stimuli appear along the 0°/180° meridian.

The events in the attention breadth conditions were similar to the Control condition except that the stimuli were displayed in pairs (see Figure 2), the stimuli remained visible for 300 ms rather than 150 ms, and subjects were prompted to report the number of light gray triangles first in one, and then in the other stimulus (selected randomly). A correct response required an accurate report of the number of light gray triangles in both stimuli. In the Fixate Center condition, the fixation cross appeared in the display center and the two pre-cues appeared equidistant from and on opposite sides of fixation along one of the meridians. These pre-cues indicated the future positions of the two peripheral stimuli. Subjects were instructed to focus their gaze on the fixation cross and to report the number of light gray triangles in each target.

In the Fixate Target condition (one fixated and one peripheral stimulus), the positions of the target stimuli were identical to those in the Fixate Center condition, but participants fixated one of the targets rather than the center of the screen. Each trial began with a fixation cross at the location where one of the two stimuli would appear (selected randomly). Subjects were instructed to fixate that location. A pre-cue circle then indicated the location where the other target would appear. After the pre-cue, both targets appeared, one at fixation and one in the periphery. Thus, the only difference between the two attention breadth conditions was where participants fixated.

Although the two stimuli were equally separated from each other (at distance ∂) in the two attention breadth conditions, because subjects fixated different display locations, the distance of each object from fixation varied across conditions. In the Fixate Target condition, one stimulus was at fixation and the other was at a distance of ∂ from fixation. In the Fixate Center condition, each stimulus was 0.5*∂ from fixation (on opposite sides of fixation).

### Data Collection

Eye position was monitored with a mobile, video-based eye tracking system (Mobile Eye®, Applied Science Laboratories, Bedford, USA) at a sampling rate of 30 Hz and a resolution of 1° (or about 3 cm on the screen). When a subject failed to maintain fixation on the fixation cross at the beginning of a trial, that trial was discarded from analysis (4% of trials in experts and 3% of trials in novices for the Control condition; 2% of trials in experts and 4% in novices across the attention breadth conditions). Subjects’ verbal responses were manually keyed in by the experimenter during each trial. For the attention breadth conditions, responses were counted as correct only if the subject reported the correct number of light gray triangles in both stimuli. When only one of the two stimuli was correctly identified, the response was treated as an error.

## Results

Given that accuracy data tend not to be normally distributed, particularly with high accuracy levels, data were transformed using the arcsine of the square root prior to all analyses. The reported means and standard deviations are based on the untransformed data. In the Control condition (see Table 1), subjects consistently reported the correct number of light gray triangles (*M* = 98.47%, *SD* = 1.09%), with comparable performance by experts (*M* = 98.33%, *SD* = 1.12%) and novices (*M* = 98.61%, *SD* = 1.11%). We conducted a 2×3×4 (Expertise [expert, novice] ×Meridian [horizontal, vertical, diagonal] ×Distance from Fixation [4°, 8°, 12°, 16°]) analysis of variance (ANOVA). Since Mauchly’s test revealed violations of the sphericity assumption for both the Distance from Fixation, χ^2^(5) = 14.293, *p* = .014, and Meridian, χ^2^(2) = 6.773, *p* = .034, factors, we used adjusted degrees of freedom based on the Greenhouse-Geisser correction. For these and other analyses in which the sphericity assumption was violated, we reported the value of ε from the Greenhouse-Geisser correction.

**Table 1:** Mean (standard deviation) percentage of correct responses for experts and novices in the Control condition.

	Stimulus Distance from Fixation											
	Horizontal				Vertical				Diagonal			
	4°	8°	12°	16°	4°	8°	12°	16°	4°	8°	12°	16°
Experts	98.75 (2.64)	97.50 (3.23)	97.50 (3.23)	98.75 (2.64)	100.00 (0.00)	98.75 (2.64)	98.75 (2.64)	97.50 (3.23)	98.13 (3.02)	99.38 (1.98)	97.50 (3.23)	97.50 (3.23)
Novices	98.61 (2.76)	97.92 (3.13)	97.92 (3.13)	97.22 (3.29)	98.61 (4.17)	97.92 (3.13)	99.31 (2.08)	99.31 (2.08)	98.61 (2.76)	98.62 (2.76)	99.31 (2.08)	100.00 (0.00)

The ANOVA revealed no significant main effects (Distance from Fixation: *F*(1.9, 31.8) = 1.539, *p* = .231, η^2^ = .083, ε = .624; Meridian: *F*(1.5, 25.3) = 1.117, *p* = .327, η^2^ = .062, ε = .743; Expertise: *F*(1, 17) = 0.393, *p* = .539, η^2^ = .023) and no significant interactions (Meridian×Expertise: *F*(2, 34) = 0.634, *p* = .537, η^2^ = .036; Distance from Fixation×Expertise: *F*(3, 51) = 3.353, *p* = .026, η^2^ = .165; Meridian×Distance from Fixation: *F*(6, 102) = 0.542, *p* = .775, η^2^ = .031; Expertise×Meridian×Distance from Fixation: *F*(6, 102) = 0.985, *p* = .439, η^2^ = .055).

**Figure 3:**
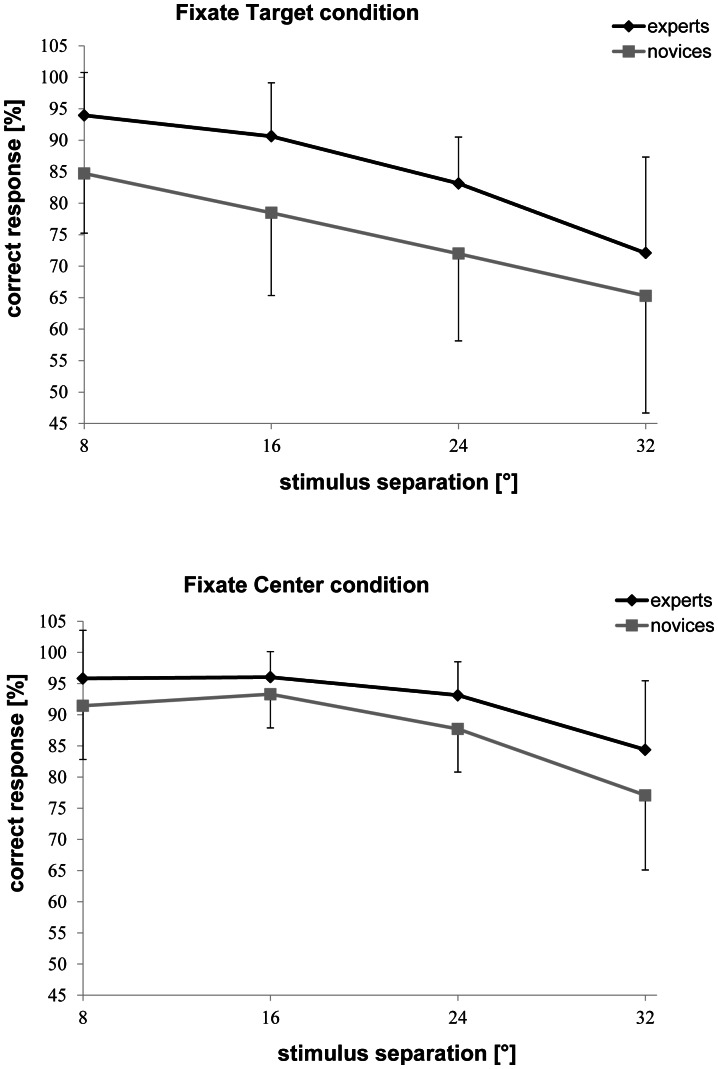
Percentage of correct responses in the Fixate Target condition (top panel) and the Fixate Center condition (bottom panel) for experts and novices as a function of stimulus separation. Symbols represent across-subject means, and error bars show standard deviations.

Accuracy rates in the attention breadth task were submitted to a 2×2×3×4 (Expertise [expert, novice] ×Condition [Fixate Center, Fixate Target] ×Meridian [horizontal, vertical, diagonal] ×Stimulus Separation [8°, 16°, 24°, 32°]) ANOVA. Overall, subjects were more accurate in the Fixate Center condition (*M* = 89.99%, *SD* = 5.13%) than in the Fixate Target condition (*M* = 80.29%, *SD* = 10.26%), *F*(1, 17) = 15.064, *p* = .001, η^2^ = .470 (see Table 2). Experts (*M* = 88.65%, *SD* = 3.37%) outperformed novices (*M* = 81.25%, *SD* = 5.33%), *F*(1, 17) = 12.900, *p* = .002, η^2^ = .431, but Expertise did not interact significantly with the Condition, *F*(1, 17) = 0.279, *p* = .604, η^2^ = .016: Experts outperformed novices in both the Fixate Target (experts: *M* = 84.95%, *SD* = 6.25%; novices: *M* = 75.12%, *SD* = 11.66%, see Figure 3, top), *F*(1, 17) = 5.143, *p* = .037, η^2^ = .232, and the Fixate Center condition (experts: *M* = 92.34%, *SD* = 3.66%; novices: *M* = 87.38%, *SD* = 5.45%, see Figure 3, bottom), *F*(1, 17) = 5.973, *p* = .026, η^2^ = .260.

**Table 2:** Mean (standard deviation) percentage of correct responses for experts and novices in the Fixate Target and the Fixate Center condition.

		Stimulus Separation											
		Horizontal				Vertical				Diagonal			
		8°	16°	24°	32°	8°	16°	24°	32°	8°	16°	24°	32°
**Fixate Target condition**													
Experts	100.00(0.00)		91.88 (10.64)	90.00(9.86)	73.75 (18.35)	97.50 (4.37)	93.13(9.97)	78.13 (23.25)	75.00 (19.54)	84.38 (20.47)	86.88 (11.95)	81.25(7.22)	67.50 (12.08)
Novices	86.11(18.43)		84.03 (19.79)	77.08 (19.26)	70.83 (23.59)	89.58 (15.93)	80.56 (15.45)	74.31 (18.60)	62.50 (24.00)	78.47 (15.34)	70.83 (13.98)	64.58 (12.50)	62.50 (13.62)
**Fixate Center condition**													
Experts	98.75 (2.64)		96.88 (4.42)	98.13 (3.02)	91.88 (5.15)	98.75 (3.95)	98.12 (3.02)	93.75 (8.33)	83.13 (19.11)	90.00 (23.61)	93.13 (9.97)	87.50 (16.40)	78.13 (18.22)
Novices	96.53 (4.54)		97.22 (4.54)	95.14 (5.21)	84.72 (10.42)	96.53 (3.29)	97.22 (4.54)	90.97 (5.51)	73.61 (20.91)	81.25 (25.58)	85.42 (15.93)	77.08 (20.49)	72.92 (12.88)

Across Meridian, Condition, and Expertise, subjects correctly performed better for stimuli separated by 8° (*M* = 91.67%, *SD* = 7.01%), than by 16° (*M* = 89.80%, *SD* = 6.59%), 24° (*M* = 84.21%, *SD* = 6.26%), or 32° (*M* = 74.89%, *SD* = 12.87%), *F*(1.5, 25.0) = 22.790, *p*<.001, η^2^ = .573, ε = .489. Expertise did not interact significantly with Stimulus Separation, *F*(3, 51) = 0.135, *p* = .939, η^2^ = .008: Experts outperformed novices in all of the four stimulus separations (see Figure 3). Across Condition, Stimulus Separation, and Expertise, subjects were more accurate along the horizontal meridian (*M* = 89.72%, *SD* = 8.01%), than along the vertical (*M* = 86.60%, *SD* = 9.40%), or the diagonal meridian (*M* = 79.11%, *SD* = 10.87%), *F*(1.2, 20.7) = 6.840, *p* = .012, η^2^ = .287, ε = .608. The effect of Meridian did not significantly interact with Expertise, *F*(2, 34) = 0.116, *p* = .891, η^2^ = .007: Experts outperformed novices along all of the three meridians (see Table 2). Furthermore, the effect of Meridian did not interact with Stimulus Separation, *F*(6, 102) = 1.447, *p* = .204, η^2^ = .078, or Condition, *F*(2, 34) = 0.098, *p* = .907, η^2^ = .006. However, the Stimulus Separation×Condition interaction was significant, *F*(3, 51) = 5.128, *p* = .004, η^2^ = .232: Accuracy decreased more in the Fixate Target Condition than in the Fixate Center condition with greater stimulus separations (cf. Figure 3).

Neither of the three-way interactions (Stimulus Separation×Meridian×Expertise: *F*(6, 102) = 1.579, *p* = .161, η^2^ = .085; Stimulus Separation×Condition×Expertise: *F*(3, 51) = 2.648, *p* = .059, η^2^ = .135; Meridian×Condition×Expertise: *F*(2, 34) = 0.326, *p* = .724, η^2^ = .019; Stimulus Separation×Meridian×Condition: *F*(6, 102) = 0.480, *p* = .822, η^2^ = .027), nor the four-way interaction were significant, *F*(6, 102) = 0.865, *p* = .523, η^2^ = .048.

In the Fixate Target condition, participants might prioritize attention to the stimulus at fixation over the one in the periphery. Consistent with that possibility, performance was more accurate for the fixated stimulus (*M* = 91.89%, *SD* = 3.57%) than for peripheral stimuli (*M* = 81.36%, *SD* = 9.44%), *F*(1, 17) = 114.385, *p*<.001, η^2^ = .871. This pattern did not significantly interact with the level of Expertise, *F*(1, 17) = 3.556, *p* = .077, η^2^ = .173. The overall effect of Expertise was marginally significant, *F*(1, 17) = 3.952, *p* = .063, η^2^ = .189.

In the Control condition, accuracy was greater than 97% for all distances and did not decrease linearly with distance (e.g., performance was more accurate at 12° than at 8°). Performance was consistently lower for all distances in the attention breadth conditions than in the Control condition, and the effects of distance were more pronounced in the attention breadth conditions. Consequently, the effects of distance in the attention breadth conditions can be attributed mostly to the increased attention demands rather than to differential acuity as a function of eccentricity.

Given that the distance of each stimulus to fixation was different in the two attention breadth conditions, we conducted a 2×2×2×3 (Expertise [expert, novice] ×Condition [Fixate Center, Fixate Target] ×Actual Distance from Fixation [8°, 16°]×Meridian [horizontal, vertical, diagonal]) ANOVA to compare performance for the peripheral stimuli in the Fixate Target condition to equally distant peripheral stimuli in the Fixate Center condition. For this analysis, a stimulus distance of 16° in the Fixate Center condition was treated as equivalent to a stimulus distance of 8° in the Fixate Target condition, and stimulus distance of 32° in the Fixate Center condition as equivalent to stimulus distance of 16° in the Fixate Target condition.

The ANOVA revealed a significant main effect for Actual Distance from Fixation, *F*(1, 17) = 32.201, *p*<.001, η^2^ = .654: Overall, subjects correctly identified more stimuli at 8° distance from fixation (*M* = 92.16%, *SD* = 5.59%) than at 16° distance (*M* = 82.90%, *SD* = 8.59%). Participants were more accurate along the horizontal meridian (*M* = 91.78%, *SD* = 7.95%), than along the vertical (*M* = 89.31%, *SD* = 8.89%), or the diagonal meridian (*M* = 81.50%, *SD* = 11.04%), *F*(1.3, 21.9) = 7.969, *p* = .006, η^2^ = .319, ε = .643 (see Table 3). Subjects showed comparable performance in the Fixate Center (*M* = 87.83%, *SD* = 6.14%) and in the Fixate Target condition (*M* = 87.23%, *SD* = 10.10%), *F*(1, 17) = 0.002, *p* = .962, η^2^<.001. And, overall, experts (*M* = 91.25%, *SD* = 3.29%) outperformed novices (*M* = 83.39%, *SD* = 5.85%), *F*(1, 17) = 11.569, *p* = .003, η^2^ = .405. Expertise did not interact significantly with Condition, *F*(1, 17) = 1.333, *p* = .264, η^2^ = .073, Actual Distance from Fixation, *F*(1, 17) = 0.475, *p* = .500, η^2^ = .027, or Meridian, *F*(2, 34) = 0.083, *p* = .921, η^2^ = .005.

**Table 3:** Mean (standard deviation) percentage of correct responses for both conditions with stimuli at the same actual distance from fixation.

	Actual Stimulus Distance from Fixation					
	Horizontal		Vertical		Diagonal	
	8°	16°	8°	16°	8°	16°
**Fixate Target condition**						
Experts	100.00 (0.00)	91.88 (10.64)	97.50 (4.37)	93.13 (9.97)	84.38 (20.47)	86.88 (11.95)
Novices	86.11 (18.43)	84.03 (19.79)	89.58 (15.93)	80.56 (15.45)	78.47 (15.34)	70.83 (13.98)
**Fixate Center condition**						
Experts	96.88 (4.42)	91.88 (5.15)	98.13 (3.02)	83.13 (19.11)	93.13 (9.97)	78.13 (18.22)
Novices	97.22 (4.54)	84.72 (10.42)	97.22 (4.54)	73.61 (20.91)	85.42 (15.93)	72.92 (12.88)

Averaging across levels of Expertise and Meridian, the interaction of Condition and Actual Distance from Fixation was significant, *F*(1, 17) = 4.896, *p* = .041, η^2^ = .224: Performance in the Fixate Center condition (*M* = 94.74%, *SD* = 4.83%) marginally exceeded that in the Fixate Target condition (*M* = 89.58%, *SD* = 9.27%) with stimuli 8° from fixation, *F*(1, 17) = 3.722, *p* = .071, η^2^ = .180, but did not for stimuli 16° from fixation (Fixate Center: *M* = 80.92%, *SD* = 11.79%; Fixate Target: *M* = 84.87%, *SD* = 12.32%), *F*(1, 17) = 1.270, *p* = .275, η^2^ = .070. Neither the Distance×Meridian interaction, *F*(2, 34) = 2.139, *p* = .133, η^2^ = .112, nor the Meridian×Condition interaction, *F*(2, 34) = 0.397, *p* = .675, η^2^ = .023, was significant. The three- and four-way interactions were not statistically significant (Actual Distance from Fixation×Meridian×Expertise: *F*(2, 34) = 1.959, *p* = .157, η^2^ = .103; Distance×Condition×Expertise: *F*(1, 17) = 0.302, *p* = .590, η^2^ = .017; Meridian×Condition×Expertise: *F*(2, 34) = 0.213, *p* = .809, η^2^ = .012; Distance×Meridian×Condition: *F*(2, 34) = 0.466, *p* = .631, η^2^ = .027; Distance×Meridian×Condition×Expertise: *F*(2, 34) = 2.564, *p* = .092, η^2^ = .131).

## Discussion

In many real-world tasks, observers must devote attention simultaneously to multiple objects or events. For example, a defending basketball player must focus attention both on the ball and on the opposing player [9], players taking a penalty shot in soccer must attend both to the goalkeeper and the ball [30], and drivers must attend to traffic from both directions. Yet, few experiments have explored the impact of different gaze strategies on performance when people must attend simultaneously to multiple objects; most extant studies deal only with the case in which people fixate one target and process another in the periphery (e.g., [10]). To our knowledge, this study is the first to compare that strategy with a comparable one in which subjects fixate midway between two objects. We found better performance when subjects fixated between the targets, a benefit that seems to result in part from the difference in eccentricity required by the two strategies: Fixating one target means that the other must be attended at a greater visual eccentricity. The advantage gained by fixating one target is offset by an even larger cost to attending to the second stimulus further into the periphery. Consistent with the claim that both conditions measure attention breadth, expert athletes, a group that should have better attention breadth (e.g., [26]), outperformed a group of novice athletes.

Inferior performance in the Fixate Center condition could also result from the tendency to prioritize any stimulus at fixation relative to stimuli in the periphery or from a difference in the absolute direction of gaze. In the Fixate Center condition, observers fixated the center of the screen. In contrast, in the Fixate Target condition, gaze was directed off-center to one of the stimulus locations. This difference was necessary in order to compare performance with each strategy using identical stimuli, but absolute gaze direction could also affect attention breadth. Future studies could explore the effect of absolute gaze position on attention breadth by using a Fixate Center strategy but varying the fixation location relative to the center of the screen.

Our design does not permit us to determine whether participants attended simultaneously to both stimuli or shifted attention rapidly from one stimulus to the other (e.g., [31], [32]). However, there is reason to think that the attention shifting account is less plausible given our findings. Presumably, in the Fixate Target condition, participants would attend first to the fixated target and then shift attention to the peripheral target, a total attention shift of distance ∂. In the Fixate Center condition, participants would first have to shift attention to one stimulus and then shift to the other, for a total distance of 1.5*∂. Moreover, they would have to decide which stimulus to shift to first and then they would have to initiate a second shift. Given the need for multiple shifts and the greater distance for attention to travel, the attention shifting account should predict worse performance rather than better performance in the Fixate Center condition.

In the Control condition, performance was near ceiling for all distances, and did not decline consistently with increasing distance. Consequently, performance in the attention breadth conditions as a function of distance was limited more by the resolution of attention than by acuity limits (e.g., [33], [34]). In both attention breadth conditions, performance was substantially worse than in the Control condition and decreased with increasing stimulus distance, consistent with the idea that spreading attention more widely reduces processing efficiency [35].

The standard method for assessing attention breadth–the Useful Field of View (UFOV) task [36], [13]–is conceptually similar to our Fixate Target condition, except that our central and peripheral tasks are identical feature conjunction tasks (the UFOV uses a detection task in the periphery). Attention breadth as measured by the UFOV predicts impaired driving in old age (e.g., [14], [15], [16], [17], [18], [19], [20]). Given the better performance achieved with a Fixate Center strategy, supplementing the UFOV with such a measure might enhance its predictive validity. Moreover, if people default to a strategy of fixating one target and attending to the other peripherally, then training them to fixate between targets could potentially improve their performance. If effective, such fixation training could have wide ranging practical implications, including compensating for any narrowing of the attention window or enhancing the performance of athletes and referees [37], [38], [39], [40]. Future studies should explore whether people fixate one of two objects by default, and subsequent training studies could evaluate the causal efficacy of practice in switching fixation strategies.

The relative efficacy of processing one or both objects peripherally likely varies with the demands of the situation. When both stimuli demand comparable amounts of attention and similar levels of acuity, fixating between them may produce better performance. That strategy assumes that both can be processed in the periphery. In contrast, when one stimulus demands greater acuity or effort than the other, fixating the more critical one may lead to better performance.

Our comparison of sport experts and novices helps to validate our measures of attention breadth. Consistent with evidence that expert athletes generally do not differ from novices in basic measures of visual perception [41], [42], [43], [44], experts and novices performed comparably well in our Control condition. This finding confirms that there was not a pre-existing group difference in visual acuity. Studies of athletes often do find expert/novice differences for attention-demanding tasks with multiple objects (e.g., [45], [46], [47], [48], [49]). The present study confirms these finding by showing better attention performance by athletes when focusing attention simultaneously on two stimuli.

In sum, we introduced a new measure of attention breadth that assesses performance when people fixate between two equally attention-demanding targets and process both peripherally. Traditional measures of attention breadth require people to fixate one target and process the other peripherally, but such tasks might underestimate performance and attention breadth when fixating between two objects is a viable strategy. When people must fixate two objects and give each equal priority, the strategy of fixating between the objects leads to better performance, raising the possibility that applying this fixation strategy could enhance performance across a range of real-world tasks that require simultaneous attention to multiple objects.
